# Androgen receptor dynamics in prostate cancer: from disease progression to treatment resistance

**DOI:** 10.3389/fonc.2025.1542811

**Published:** 2025-02-11

**Authors:** Caihong Li, Dongkai Cheng, Peng Li

**Affiliations:** Center for Reproductive Medicine, Shenyang Jinghua Hospital, Shenyang, China

**Keywords:** prostate cancer, androgen receptor (AR), epigenetics, drug resistance, personalized therapy

## Abstract

Prostate cancer is the most common cancer among men worldwide, especially in those over 65, and is a leading cause of cancer-related mortality. The disease typically advances from an androgen-dependent state to castration-resistant prostate cancer (CRPC), which poses significant treatment challenges. The androgen receptor (AR) on the X chromosome is a central driver in this process, activating genes that govern proliferation and survival. Mutations and amplifications of the AR are closely associated with disease progression and treatment resistance. While traditional therapies such as androgen deprivation therapy (ADT) and AR antagonists like enzalutamide have been effective, resistance persists due to reactivation of AR signaling through mechanisms like ligand-independent activation. Recent research highlights the role of epigenetic modifications in enhancing AR activity and drug resistance. The tumor microenvironment, particularly interactions with cancer-associated fibroblasts (CAFs) and tumor-associated macrophages (TAMs), further complicates treatment by promoting aggressive tumor behavior and immune evasion. Future directions include developing next-generation AR antagonists, identifying AR-related biomarkers for personalized therapy, and exploring combinations with immune checkpoint inhibitors. Additionally, basal cell-lumen-derived organoids provide innovative models that can enhance understanding and treatment strategies in prostate cancer.

## 1 Introduction

Prostate cancer is the second most common cancer type among men globally, accounting for 7.3% of all new cancer cases and ranking as one of the leading causes of cancer-related death in males, coming in fifth place (1). Its incidence significantly increases with age, particularly among men over 65 years old (2). Studies show that prostate cancer is a highly heterogeneous disease, with its progression typically evolving from androgen-dependent cancer to castration-resistant prostate cancer (CRPC), which is the most difficult stage of the disease to treat (3, 4).

The androgen receptor (AR) is a central driving force in the incidence and progression of prostate cancer. It activates the expression of related genes by binding to androgens (such as testosterone and dihydrotestosterone), thereby regulating cell proliferation, differentiation, and survival (5). The AR protein consists of 919 amino acids and can be structurally divided into four domains: the N-terminal domain (NTD), which is responsible for transcriptional activation; the DNA-binding domain (DBD), which mediates gene-specific binding; the hinge region (which regulates nuclear localization and stability); and the ligand-binding domain (LBD), which binds to androgens (6). The AR gene is located on the X chromosome and consists of eight exons; mutations or amplifications of this gene are often closely associated with the progression of prostate cancer and treatment resistance (7–9).

Traditional treatment strategies focus on blocking the binding of AR to androgens or inhibiting the transcriptional activity of AR, including androgen deprivation therapy (ADT) and second-generation AR antagonists (such as enzalutamide and apalutamide) (10). These treatment methods have significantly extended the progression-free survival of patients. However, the issue of resistance remains a major clinical challenge. Research indicates that the reactivation of AR signaling is a primary cause of CRPC development, with mechanisms including AR gene amplification or mutation, ligand-independent activation, bypass signaling pathway compensation, and the production of AR splice variants (such as AR-V7) (11, 12). Notably, AR-V7 is a variant that lacks the ligand-binding domain and can activate downstream signaling pathways even in the absence of androgens, making it a critical driver of CRPC resistance (13).

In recent years, studies in epigenetics have provided new perspectives for understanding the regulatory mechanisms of AR and drug resistance (14, 15). Research by Nguyen et al. found that H2AK130ac is enriched in the promoter regions of key androgen synthesis genes (such as CYP17A1) in prostate cancer cells, correlating with their high expression levels (16). Additionally, RNA modifications (such as m6A methylation) have also been shown to play a significant role in the stability and translation efficiency of AR mRNA, providing a foundation for potential therapeutic targets (17, 18). More importantly, by integrating multi-omics technologies, researchers are attempting to develop novel combination therapy strategies that target key nodes in the AR network, such as the co-inhibition of AR signaling and the PI3K/Akt pathway (19).

In CRPC, specific epigenetic changes play a crucial role in the reactivation of AR signaling (20). DNA methylation changes, particularly hypomethylation in the promoter regions of AR target genes, can lead to increased gene expression, facilitating AR reactivation even during androgen-deprivation therapy. Histone modifications, such as altered acetylation and methylation, can modify chromatin accessibility, influencing AR transcriptional activity. Increased histone acetylation at AR target sites, for instance, can enhance AR binding and transcriptional activation, promoting cancer progression despite low androgen levels. Moreover, non-coding RNAs, including microRNAs and long non-coding RNAs (lncRNAs), can regulate AR expression and activity by interacting with AR mRNA or influencing chromatin states, contributing further to AR reactivation in CRPC.

Targeting histone modifications or RNA methylation pathways holds promise but presents challenges due to the genetic heterogeneity of CRPC (21). The effectiveness of such approaches can vary across different genetic profiles, as certain modifications may be more prevalent or impactful in specific genetic contexts. While drugs targeting histone modifiers, such as Histone Deacetylase(HDAC) or Histone Methyltransferase(HMT) inhibitors, or RNA methylation processes could have broader applications, their success largely depends on identifying patient subgroups most likely to benefit from these treatments (22). This underscores the potential for employing personalized strategies that integrate genomic and epigenomic data to enhance treatment efficacy. Additionally, the possibility of resistance necessitates the exploration of combination therapies that simultaneously target multiple pathways involved in AR reactivation and CRPC progression.

In summary, as a core molecular target in prostate cancer, AR plays an indispensable role in the occurrence, progression, and treatment resistance of the disease. In-depth research into the regulatory mechanisms of AR signaling, combined with the latest findings from molecular biology and epigenetics, can enhance our understanding of the mechanisms underlying CRPC development and provide new directions and ideas for its diagnosis and treatment. This article will focus on the molecular regulatory mechanisms of AR and the latest research progress in prostate cancer treatment.

## 2 Androgen receptor signaling pathway

Androgens bind to the LBD of AR, inducing a conformational change that enables AR to translocate from the cytoplasm to the nucleus with the assistance of chaperone proteins, such as heat shock proteins (HSPs). These chaperones stabilize AR and facilitate its interaction with cofactors during nuclear import. Once in the nucleus, AR dimerizes and binds to androgen response elements (AREs) in the promoter regions of target genes, promoting their transcription (23).

**Figure 1:**
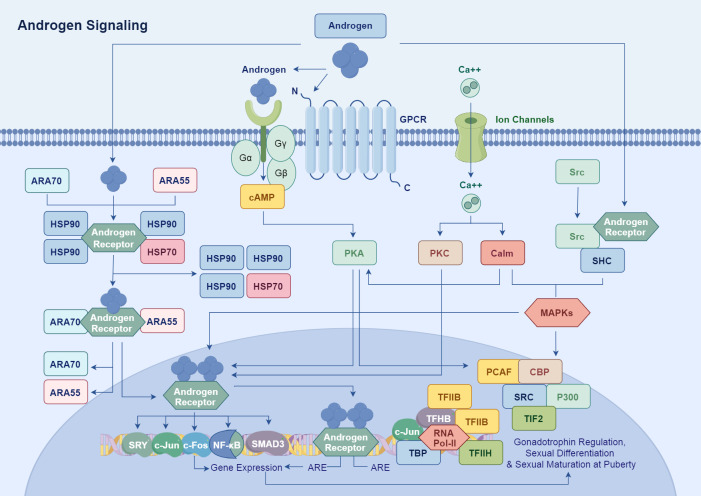
Androgens bind to the LBD of AR, inducing conformational changes in AR. This process allows AR to translocate from the cytoplasm to the nucleus, assisted by chaperone proteins. In the nucleus, AR forms a dimer and binds to ARE in the promoter regions of target genes, thereby promoting their transcription. AR enhances the expression of genes related to cell growth, differentiation, apoptosis, and metabolism by recruiting transcriptional co-factors such as the SRC family and CBP/p300 to form transcriptional complexes.

AR enhances the expression of genes associated with cell growth, differentiation, apoptosis, and metabolism by recruiting transcription co-factors, such as members of the SRC family and CBP/p300, which form transcriptional complexes that enhance AR-mediated transcriptional activity (24, 25). Additionally, AR synergistically interacts with transcription factors like c-Jun, NF-κB, and SMAD3, alongside cofactors such as CBP and PCAF, to modulate the expression of key genes involved in sexual differentiation (e.g., SRY), gonadal function, and pubertal maturation (**Figure 1**) (26). For example, Cyclin D1 and Cyclin E are upregulated by AR, facilitating cell cycle progression by promoting the G1 to S phase transition. Moreover, AR regulates the expression of anti-apoptotic factors, such as Bcl-2 and Bcl-xL, while inhibiting pro-apoptotic genes like P53, thereby ensuring continuous cell survival in normal and malignant cells (27).

Androgen signaling also amplifies cellular responses through interactions with other signaling pathways, including MAPK and Src, thereby achieving comprehensive regulation of gonadal function, sexual differentiation, and pubertal maturation (28). Specifically, AR has been shown to interact with growth factor receptors like EGFR and TrkA, as well as with metalloproteases in prostate cancer, which can influence AR signaling dynamics and enhance tumor progression (29–32).

Understanding the nuances of AR signaling is critical, particularly regarding how the modulation of Cyclin D1 and Bcl-2 contributes to prostate cancer development and progression. These factors are essential, as Cyclin D1 is pivotal for cell cycle regulation, while Bcl-2 plays a crucial role in promoting cell survival, thus highlighting the importance of aberrant AR signaling in the pathology of prostate cancer.

## 3 Relationship between androgen receptor and prostate cancer

Androgens are important factors for the growth of prostate cancer cells, and prostate cancer typically develops under the influence of androgen signaling. In the early stages, once androgens bind to AR, AR translocates to the nucleus and binds to specific enhancer elements, initiating the transcription of a series of genes related to cell proliferation and survival, including MYC and KLK3 (prostate-specific antigen) (33). The activation of this AR signaling pathway effectively promotes the growth of prostate cancer cells.

As the disease progresses, the expression levels and function of AR undergo significant changes, usually leading to a transition from an androgen-dependent state to an androgen-independent state (34). Throughout the development of prostate cancer, AR expression levels tend to gradually increase, particularly in CRPC, where the expression of AR protein significantly rises. This increase in expression is viewed as an adaptive survival strategy of tumor cells in a low-androgen environment (35).

Studies have shown that compared to hormone-sensitive prostate cancer, AR expression is notably upregulated in CRPC tissues, and this change is closely associated with enhanced proliferation capacity of tumor cells and treatment resistance (36). The elevated AR expression enables tumor cells to remain sensitive to low concentrations of androgens, maintaining the activity of the AR signaling pathway even in conditions of extremely low exogenous androgen supply. This mechanism plays a critical role in AR-mediated tumor signaling, further driving the malignant progression of the tumor.

## 4 The role of AR in prostate cancer-associated tumor microenvironment

AR not only influences the growth of tumor cells themselves but also affects tumor development and progression by regulating immune cell responses in the tumor microenvironment (37). Changes in the microenvironment are primarily reflected in several aspects:

First, tumor cells can secrete various cytokines (such as IL-6, IL-10, and TNF-α), and the changes in the expression of these cytokines not only affect the infiltration and activity of leukocytes but also promote alterations in the biochemical properties of surrounding fibroblasts. Second, tumor cells can induce an immunosuppressive microenvironment, making it difficult for the immune system to effectively attack the tumor (38). Tumor-associated macrophages (TAMs) typically exhibit characteristics that suppress T cell activity (39). Additionally, the interactions between tumor cells and the surrounding stroma enhance the invasive ability of the tumor, and changes in the stroma are also key factors in tumor metastasis.

TAMs and other immune components significantly contribute to treatment resistance in cancer by interacting with the AR (40). TAMs secrete various pro-inflammatory cytokines and growth factors, such as IL-6 and IGFs, which can activate the AR signaling pathway, promoting cancer cell growth and enhancing resistance to therapies, including antiandrogen treatment. Besides, TAMs and other immunosuppressive cells, such as myeloid-derived suppressor cells, create an immunosuppressive microenvironment that diminishes the effectiveness of antitumor immune responses, enabling tumor cells to evade control and develop resistance to treatments. Modulating cytokines like IL-6 and TNF-α could potentially shift the tumor microenvironment to support AR-targeting therapies. By regulating these cytokines, the tumor-promoting activities of TAMs might be reduced, and antitumor immune responses could be restored. Moreover, combining cytokine inhibitors with AR-targeting therapies could enhance their efficacy by mitigating the pro-inflammatory and immunosuppressive effects of the cytokines. Such modulation may also improve immune cell functionality, thus enhancing their antitumor activity and synergistically increasing the effectiveness of AR-targeting therapies.

AR also interacts with signaling pathways such as PI3K/AKT/mTOR, Wnt/β-catenin, and NF-κB, regulating cell proliferation and survival. Among these, the PI3K/AKT/mTOR signaling pathway plays a crucial role in promoting survival in prostate cancer, with its activation often accompanied by the loss of PTEN. Research indicates that there is a bidirectional regulation between the PI3K/AKT/mTOR pathway and AR signaling. Carver et al. found in mouse models that the activation of the PI3K pathway can inhibit AR expression through negative feedback, while inhibition of the PI3K pathway can upregulate AR expression (19). Furthermore, the PI3K/AKT signaling pathway can enhance the transcriptional activity of the androgen receptor, promoting tumor cell dependence on AR signaling. This reciprocal regulatory mechanism may be an important reason for treatment resistance in CRPC.

The Wnt/β-catenin signaling pathway is considered a key pro-cancer signal in prostate cancer that can interact synergistically with AR signaling to promote tumor cell proliferation and metastasis. A study by Yuan et al. demonstrated that Wnt signaling activation can enhance the interaction between β-catenin and AR by stabilizing β-catenin, thereby increasing AR’s transcriptional activity on downstream genes (41). This synergistic effect not only drives tumor cell proliferation but may also increase treatment resistance by enhancing tumor heterogeneity.

The NF-κB signaling pathway is an important regulatory factor of inflammation in the tumor microenvironment and can interact with AR signaling through various mechanisms. Thapa et al. found that NF-κB signaling activation not only directly upregulates the expression of the AR gene but can also enhance AR’s transcriptional activity by inducing the expression of co-factors that bind to AR, such as p300 (42). This interaction is particularly significant in the tumor inflammatory environment and may promote disease progression by enhancing the survival capacity of tumor cells and their immune evasion mechanisms.

Perineural invasion (PNI) is a common feature of prostate cancer, particularly evident in more aggressive tumors (43). The presence of PNI is typically associated with adverse clinical outcomes, such as an increased risk of tumor recurrence and a reduced survival rate for patients, which makes it an important prognostic marker. The microenvironment created by PNI provides favorable conditions for the survival, proliferation, and invasion of cancer cells, thereby influencing their biological behavior. Furthermore, the molecular mechanisms involved in the development of PNI primarily focus on the signaling pathways and interactions between cancer cells and nerve cells, including neurotrophic factor (NGF) and its receptors, β-adrenergic receptors, chemokines, and neural cell adhesion molecules (44). The dissemination of cancer cells around nerves is not merely a passive phenomenon; rather, it results from the complex interactions among cancer cells, nerve cells, and other components of the tumor microenvironment.

**Figure 2:**
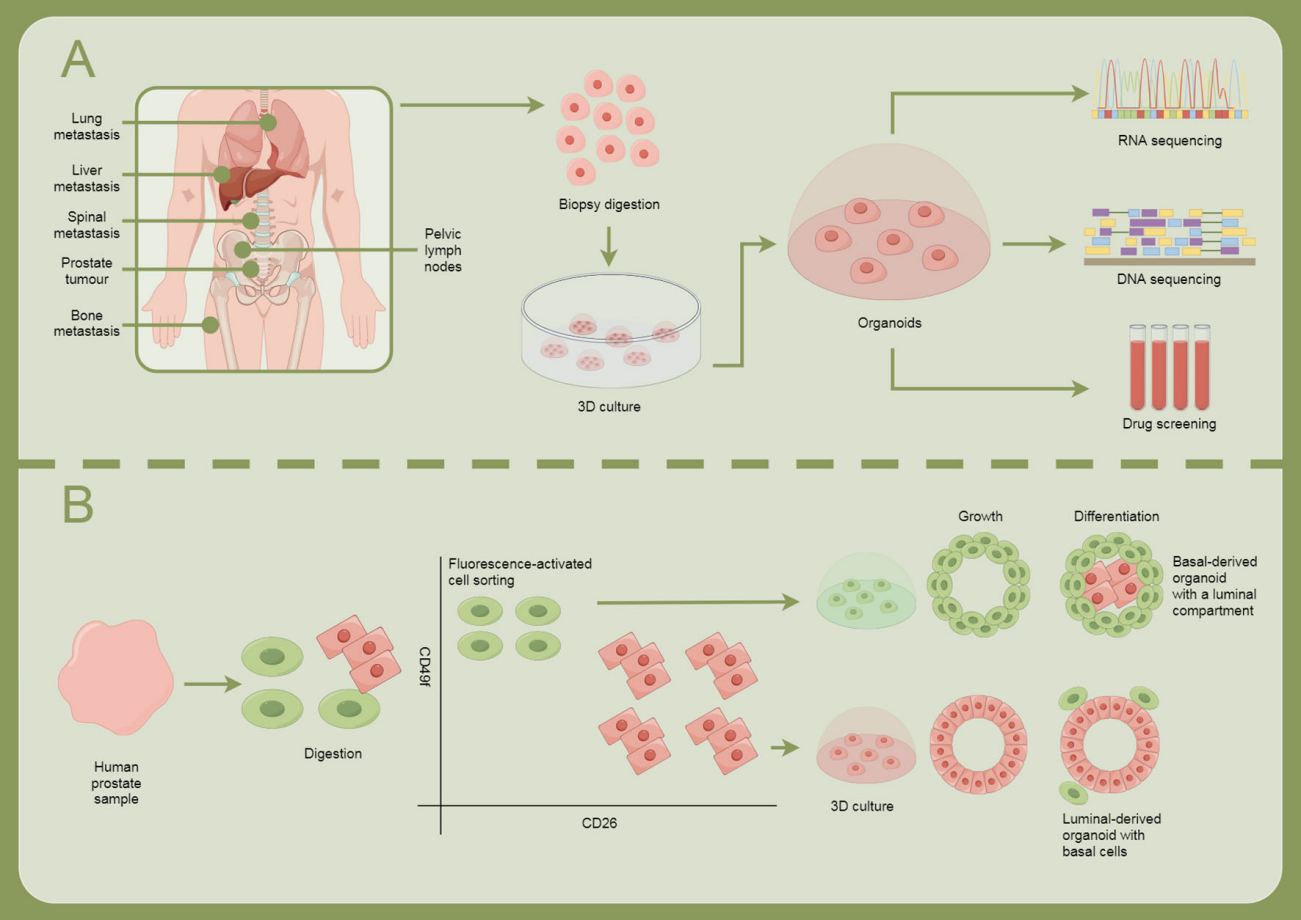
Illustrates how cellular models are used for research and treatment exploration in prostate cancer. **(A)** Prostate cancer can metastasize to sites such as the lungs, liver, spine, bones, and pelvic lymph nodes. The process involves biopsy digestion, 3D culture, organoid formation, RNA and DNA sequencing, and drug screening. **(B)** Human prostate samples are obtained, followed by digestion, fluorescence-activated cell sorting, and growth and differentiation, forming different types of organoids: basal-derived organoids with luminal structures, and luminal-derived organoids containing basal cells.

The progression of prostate cancer is significantly influenced by TME, particularly the role of cancer-associated fibroblasts (CAFs) (45). Recent studies highlight that CAFs express a transcriptionally inactive AR, which critically mediates their migration and invasiveness (46). Although androgen receptor signaling is essential for prostate cancer progression, its complex interplay with CAFs, including the binding of the AR to the scaffold protein filamin A, enhances CAF migration toward prostate cancer epithelial cells and increases tumor organoid size in both 2D and 3D cultures. This mechanism also results in extracellular matrix remodeling facilitated by a protease cascade activated through the AR/filamin A interaction and β1 integrin (47). Furthermore, androgen stimulation induces unique chromatin binding events in AR-expressing CAFs, altering cytokine production and promoting prostate cancer cell migration through paracrine signaling. Importantly, ADT affects AR expression in the stroma, contributing to the progression of the disease by dysregulating extracellular matrix protein levels and promoting a metastatic microenvironment. Disruption of the AR/filamin A complex using a stapled peptide effectively reduces CAF invasiveness and tumor growth in co-culture models, suggesting the potential for targeting this complex as a therapeutic strategy to enhance treatment outcomes in prostate cancer (48, 49). Overall, understanding the molecular cross-talk between AR signaling in CAFs and prostate cancer cells could pave the way for novel therapeutic approaches to combat drug resistance and metastatic disease in prostate cancer (**Figure 2**).

## 5 Mechanisms of androgen receptor resistance

### 5.1 AR mutations and drug resistance in prostate cancer

ADT is the mainstay treatment for metastatic prostate cancer; however, most patients eventually progress to CRPC, which exhibits poor treatment responses (50). In recent years, AR mutations have been recognized as one of the significant mechanisms underlying CRPC, primarily focusing on LBD of the androgen receptor, as this region directly participates in binding androgens or anti-androgen drugs. Various AR gene mutations are closely associated with resistance to CRPC and affect tumor cell growth and treatment response through different mechanisms (**Table 1**).

**Table 1:** The summary of androgen receptor (AR) mutation types, mutation sites, and their related therapeutic drugs.

Mutation Type	Location	Impact Description	Related Drugs
L701H	AR gene position 701	Causes AR to activate downstream genes even in the absence of androgens, often associated with resistance to androgen deprivation therapy (36).	Flutamide, Bicalutamide
T877A	AR gene position 877	Enhances AR activity in the absence of androgens, allowing prostate cancer cells to continue growing and develop resistance to treatment (41, 42).	Flutamide, Bicalutamide
F877L	AR gene position 877	Activates the AR signaling pathway by increasing the binding affinity of AR to drugs and altering its conformation post-binding (19).	Enzalutamide, Apalutamide
H874Y	AR gene position 874	Similar to T877A, may allow AR to remain active in the absence of androgens, helping tumor cells escape androgen deprivation therapy (50).	Androgen deprivation therapy
F876L	AR gene position 876	Primarily reduces the stability of antagonist binding, partially diminishing the effects of anti-androgen drugs (51).	Enzalutamide, Apalutamide
CAG Repeat Variations	AR gene first exon	Variations in CAG repeat length relate to AR activity; shorter repeats may be associated with an increased risk of prostate cancer (52).	Androgen deprivation therapy
W742C	AR gene position 742	Associated with tumor cell resistance to anti-androgen drugs (53).	Flutamide, Bicalutamide

The L701H mutation allows AR to activate downstream genes even in the absence of androgens, leading to resistance to androgen deprivation therapy (51). The T877A mutation enhances AR activity in an androgen-deprived environment, promoting the continuous growth of prostate cancer cells (51, 53). The F877L mutation alters the conformation of AR when binding with drugs, activating downstream signaling pathways and helping the tumor evade anti-androgen therapy (19). Similar to T877A, the H874Y mutation enables AR to function under androgen-deficient conditions, facilitating tumor resistance to treatment (54). The F876L mutation decreases the binding stability of anti-androgen drugs with AR, reducing the effectiveness of anti-androgen therapy (55).

Additionally, variations in the CAG repeat number in the first exon of the AR gene may also impact AR activity, with shorter CAG repeats being associated with an increased risk of prostate cancer (56). The W742C mutation is closely related to resistance to anti-androgen drugs (57). These mutation mechanisms reveal the complexity of prostate cancer treatment and provide important insights for personalized therapeutic strategies.

The existing knowledge of AR mutations, such as L701H and T877A, can be effectively utilized for precise drug design through various advanced methods. Structural biology research, including X-ray crystallography and nuclear magnetic resonance (NMR), elucidates the three-dimensional structures of AR mutants, revealing how these mutations alter the shape and charge distribution of the ligand-binding pocket, thereby affecting drug binding (58). Molecular dynamics simulations further aid in predicting optimal drug binding modes by studying the dynamic behavior of these mutants when bound to different ligands. Computer-aided drug design (CADD) is employed in virtual screening to identify candidate compounds capable of effectively targeting these mutants (59). Additionally, quantitative structure-activity relationship (QSAR) models are developed to predict the activity of new molecules on mutant ARs and optimize their chemical structures for improved efficacy (60).

High-throughput screening platforms enable the rapid identification of small molecules from large compound libraries that interact effectively with specific AR mutants. Based on screening results, directed chemical synthesis is conducted to chemically modify and optimize these molecules, enhancing their affinity and selectivity for AR mutants. Biological function validation is critical, involving testing the efficacy of candidate drugs in cell lines with specific AR mutations to assess their impact on AR activity inhibition and cancer cell proliferation. *In vivo* efficacy and safety assessments are conducted using mouse xenograft models harboring AR mutations, such as those found in CRPC.

In the context of personalized medicine, gene testing and drug matching utilize patients’ genomic information to determine specific AR mutation types, enabling the design of personalized treatment plans to select the most effective drugs. Furthermore, the identification of undiscovered AR mutations that are crucial for drug resistance is of utmost importance. In prostate cancer research, particularly in CRPC, there may be unknown AR mutations that play significant roles in drug resistance. High-throughput genomic sequencing broadens whole-genome or whole-exome sequencing to a larger patient population to uncover new AR mutations, which, although present in only a few patients, could be critical for drug resistance (61).

Functional validation of all identified mutations determines their impact on AR activity and drug resistance through *in vitro* experiments, cell line models, or animal models (62). Bioinformatics and machine learning tools are utilized to search for mutation patterns potentially associated with drug resistance from large datasets, identifying subtle yet important mutations often overlooked by traditional methods. Patient-derived models, such as organoids or xenografts, are employed for pharmacological testing to identify mutations that may lead to drug resistance, more accurately reflecting patient biological contexts. Finally, studying mutation cooperativity reveals how interactions between multiple gene mutations might also contribute to drug resistance, assisting in identifying new resistance mechanisms and addressing the challenges posed by AR mutations in prostate cancer treatment comprehensively.

### 5.2 Changes in AR downstream signaling pathways

In the mechanism of AR resistance, cellular metabolic reprogramming plays a crucial role in tumor cell adaptation to therapeutic stress, involving significant alterations in glucose metabolism, lipid metabolism, and amino acid metabolism (63). In glucose metabolism, resistant cells maintain energy supply by enhancing glycolysis while suppressing oxidative phosphorylation to adapt to oxidative stress (64). In terms of lipid metabolism, fatty acid synthesis and oxidation are activated to support rapid cell proliferation and improve antioxidant capacity. Research by Swinnen et al. has shown that AR-resistant tumor cells promote lipid synthesis by upregulating the expression of fatty acid synthase (FASN) and ATP-citrate lyase (ACL), thereby meeting the demands of rapid proliferation and membrane structure (65).

In amino acid metabolism, the reprogramming of glutamine metabolism and one-carbon metabolism provides energy, intermediate metabolites, and regulatory abilities for epigenetic modifications (66). These metabolic pathways work synergistically to support the growth, invasion, and immune evasion of resistant tumors. In the future, combining metabolomics with targeted metabolic inhibitors in combination therapy may help overcome AR resistance and optimize treatment strategies for prostate cancer.

Metabolic pathways, including lipid and amino acid metabolism, play a crucial role in sustaining AR-independent CRPC survival. CRPC cells can satisfy their energy requirements for growth and survival by enhancing fatty acid synthesis and β-oxidation, as well as altering cholesterol and other lipid metabolism to support membrane construction and signaling molecule production, enabling continued survival without androgen stimulation (67). Additionally, these cells reprogram amino acid metabolism by increasing glutamine utilization, which provides essential carbon and nitrogen for cell growth and supports antioxidant defenses to protect against oxidative stress. The potential for combined metabolic pathway inhibitors to enhance AR-targeting therapies lies in their ability to create therapeutic synergy. By inhibiting key metabolic pathways such as fatty acid synthesis or glutamine metabolism, the survival capabilities of CRPC cells can be weakened, increasing their sensitivity to AR-targeting therapies. This combination approach may effectively reduce the energy supply to tumors, thus inhibiting their growth. However, a significant challenge is reducing toxicity; it is crucial to develop highly selective metabolic inhibitors that target tumor metabolism without adversely affecting normal cell function. Optimizing dosing and administration regimens is essential to avoid unacceptable toxicity while ensuring the efficacy of the treatment.

The activation of bypass signaling pathways refers to tumor cells supporting their proliferation and survival through the activation of other growth factor signaling pathways, such as the PI3K/Akt/mTOR pathway, MAPK pathway, Wnt/β-catenin pathway, and IL-6/JAK/STAT3 pathway, even in the absence of AR or androgen signaling (68). These pathways can drive tumor growth independently of AR signaling. Carver et al. found that there is a reciprocal inhibitory relationship between AR and the PI3K/Akt/mTOR pathway; when AR is inhibited, the PI3K/Akt pathway is released and overly activated, promoting cell survival (19). Pencik et al. observed in PTEN-deficient mouse models that the loss of STAT3 led to decreased levels of LKB1 and phosphorylated AMPK (pAMPK), while simultaneously activating the mTOR/CREB pathway, accelerating the development of metastatic disease (69). In the context of AR resistance, the PI3K/Akt/mTOR, MAPK, Wnt/β-catenin, and IL-6/JAK/STAT3 pathways sustain tumor cell survival and progression through multi-layered regulation, highlighting the importance of the cooperation among multiple pathways as new therapeutic targets for metastatic prostate cancer and resistance.

Multi-omics technologies, which integrate genomic, transcriptomic, proteomic, and metabolomic data, enable the identification of key molecules and interactions within AR signaling pathways and compensatory pathways like PI3K/Akt (70). This comprehensive approach allows researchers to detect mutations, changes in gene expression, and metabolic reprogramming within these pathways, leading to the development of more precise drug targets. Additionally, by integrating multi-omics data, researchers can uncover how prostate cancer cells develop resistance to AR-targeted therapies by activating alternative pathways such as PI3K/Akt, providing crucial insights for designing more effective combination therapies. Multi-omics analysis also supports predicting patient responses to combination therapies: (1) Personalized Treatment Decisions: Multi-omics analysis provides information on the unique characteristics of each patient’s tumor, such as specific mutations, pathway activation statuses, and microenvironmental factors. This enables customized treatment strategies to enhance the efficacy of combination therapies. (2) Biomarker Development: By identifying biomarkers that predict treatment response, multi-omics technologies can help distinguish which patients are likely to respond well to specific combination therapies, aiding in more precise patient stratification before treatment. (3) Monitoring and Adjusting Treatment Efficacy: Multi-omics approaches allow for dynamic monitoring of molecular characteristics during treatment; for example, detected molecular changes can indicate rapid response or emerging resistance to therapy, enabling timely adjustments to treatment plans and improving long-term outcomes.

## 6 Treatment progress

In the treatment of CRPC, AR inhibitors represent a significant therapeutic strategy (71). These drugs inhibit AR activity through various mechanisms, significantly improving patient prognosis. Enzalutamide, a non-steroidal AR antagonist, has been approved for CRPC patients and shows survival benefits by blocking the binding of androgens to AR and inhibiting its transcriptional activity (72). Abiraterone acetate lowers the systemic levels of androgens by inhibiting the CYP17 enzyme, making it an essential treatment option for CRPC patients (73). Darolutamide, a novel AR antagonist, demonstrates good efficacy in non-metastatic CRPC (nmCRPC) and exhibits a lower incidence of central nervous system side effects due to its unique molecular structure (19). The successful development and application of these drugs have provided new treatment options for CRPC patients and established new standards for clinical treatment.

Targeting AR splice variants such as AR-V7, which evade current therapies, requires innovative strategies due to the lack of the LBD that traditional AR antagonists target (74). Key approaches focus on identifying unique structural or functional domains specific to AR-V7 to develop targeted molecular inhibitors. Additionally, technologies like small molecules, antisense RNA, or siRNA could interfere with AR-V7 mRNA processing, stability, or translation, reducing its production and activity. Another promising method is utilizing PROTAC (Proteolysis Targeting Chimeras) technology to design molecules that tag AR-V7 for protein degradation, hence lowering its cellular levels (75). However, designing AR antagonists with fewer off-target effects poses several challenges. Achieving selectivity and specificity is crucial, requiring a thorough understanding of AR’s mechanisms to selectively inhibit its functions without impacting other nuclear receptor family members. Moreover, the pharmacokinetics and metabolism of compounds must be meticulously evaluated to ensure stability, proper absorption, favorable distribution, efficient metabolism, and excretion, all while maintaining effective concentrations at target tissues with a safe profile. Finally, reducing potential toxicity and adverse effects is essential to enhance patient tolerance and ensure treatment sustainability, necessitating careful consideration of the drug design and delivery processes.

Sipuleucel-T is a dendritic cell immunotherapy vaccine targeting prostate cancer. It enhances the immune system’s response to tumor cells by increasing the binding of regulatory chemokines to prostate-specific antigen (PSA) (76). As the first dendritic cell-based prostate cancer vaccine approved by the FDA, Sipuleucel-T has shown some clinical efficacy; however, it has not provided significant breakthroughs for many advanced patients, particularly those with CRPC. To overcome the limitations of dendritic cell vaccines, current research is focusing on strategies that combine them with other immunotherapeutic agents (77). For example, the combination of immune checkpoint inhibitors with dendritic cell vaccines is being explored in clinical trials. This combined treatment not only enhances the overall immune response but also effectively overcomes immune suppression in the tumor microenvironment. In addition, adjuvants such as cytokines are being used in conjunction with dendritic cell vaccines to further improve their therapeutic efficacy (78). These multi-pronged strategies suggest that the combination of dendritic cell vaccines and immunotherapy is expected to provide more effective treatment options for prostate cancer patients in the future.

For prostate cancer patients with specific biomarkers, immune checkpoint inhibitors (such as anti-PD-1/PD-L1 antibodies, like Pembrolizumab and Nivolumab) are also undergoing clinical trials (79–81). PARP inhibitors (such as Olaparib and Rucaparib) are used for prostate cancer patients with BRCA1/2 mutations or other DNA repair deficiencies, functioning by interfering with the DNA repair mechanisms of tumor cells (82, 83). Radiopharmaceuticals (such as Radium-223) target metastatic castration-resistant prostate cancer to alleviate pain from bone metastases and extend survival (84). In addition, small molecule targeted drugs against AR and its downstream signaling pathways are also in development. Meng Wu et al. discovered and identified a bifunctional small molecule, Z15, which acts as an effective AR antagonist and a selective AR degrader. Z15 is capable of directly interacting with the LBD of AR and the activation function-1 (AF-1) region, promoting AR degradation through the proteasomal pathway (85).

## 7 Future research directions

Researchers are working to develop more efficient and selective AR antagonists to overcome the limitations of existing drugs (86). Precision-targeted therapies against AR-V7 are anticipated to significantly improve treatment outcomes for patients with CRPC, especially for those who have developed resistance to current therapies (87). Non-coding RNAs regulate the activity of the AR signaling pathway through various mechanisms, influencing the onset and progression of prostate cancer (88–92). Firstly, non-coding RNAs can directly bind to AR or regulate its downstream target genes, thereby affecting tumor cell proliferation, invasion, and resistance. Additionally, certain non-coding RNAs can mediate the expression or activity of AR variants (such as AR-V7) in CRPC, resulting in resistance to traditional AR-targeted therapies. Non-coding RNAs also indirectly influence the activity of AR signaling in the tumor microenvironment by regulating immune cell infiltration and inflammatory responses, further promoting tumor progression and resistance (93).

Research by Yaru Xu et al. indicates that Zinc Finger Protein 397 (ZNF397) is a true co-activator of AR, and Ten-eleven translocation 2(TET2) inhibitors can eliminate the resistance of ZNF397-deficient tumors to AR-targeted therapies. This mechanism reveals how prostate cancer can acquire lineage plasticity through epigenetic regulation and offers new clinical intervention strategies to overcome resistance caused by this lineage plasticity (94).

Developing personalized treatment plans based on patients’ genomic and tumor characteristics is expected to enhance treatment efficacy. In prostate cancer research, basal cell-derived organoids serve as an innovative *in vitro* culture model that effectively simulates the tissue structure and function of the prostate (95). These organoids are not only used to study tumor biology but also provide a new platform for personalized treatment and drug screening. Basal cell-derived organoids offer a highly promising experimental tool for prostate cancer research, helping scientists to better understand the biological characteristics of prostate cancer and facilitating the development and implementation of personalized treatment approaches.

**Figure 3:**
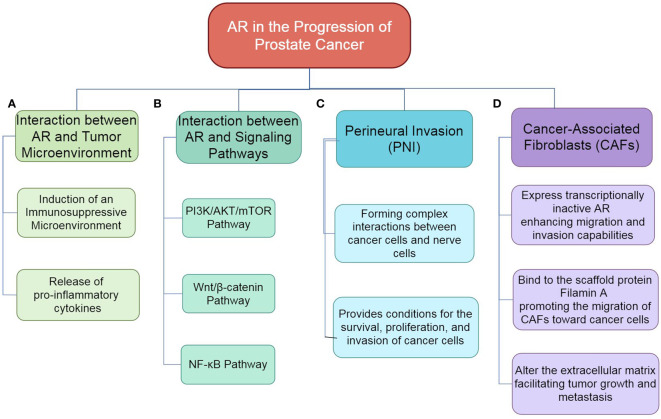
The role of AR in the progression of prostate cancer. **(A)** Interaction between AR and Tumor Microenvironment: AR influences the tumor microenvironment by mediating interactions with various stromal components, which can promote tumor growth and progression. **(B)** Interaction between AR and Signaling Pathways: AR interacts with multiple signaling pathways, including PI3K/AKT/mTOR, Wnt/β-catenin and, NF-κB, thereby modulating cellular processes such as proliferation, survival, and invasion. **(C)** PNI: AR is implicated in the process of perineural invasion, where cancer cells invade surrounding nerve tissues, contributing to tumor spread and associated pain. **(D)** Role of CAFs: AR within CAFs enhances their migratory and invasive properties, facilitating crosstalk with cancer cells and promoting a supportive microenvironment for tumor progression.

Lumen-forming basal-derived organoids represent an important three-dimensional cell culture model that better mimics the biological features of glands such as the prostate (**Figure 3**) (96). These organoids can reflect cell growth and differentiation while reproducing complex interactions within tissue structures and the microenvironment, making them particularly suitable for studying diseases like prostate cancer. As a preclinical model, basal-derived organoids have broad applications in drug screening and disease mechanism research (97). For instance, studies on personalized treatment using patient-derived organoids are continuously advancing (98). Additionally, the application of gene editing technologies like CRISPR provides convenience for functional gene studies in organoids, aiding in the in-depth understanding of tumor initiation and progression mechanisms.

Patient-derived organoids, particularly basal-derived organoids, demonstrate high accuracy in replicating patient-specific tumor responses in drug screening due to their structural and functional similarity to the original tumor tissue, preserving the genomic characteristics and heterogeneity of the patient’s tumor (99). These organoids often provide more accurate predictions of tumor responses because they can replicate complex cancer biology features, such as cell differentiation states and the influence of the tumor microenvironment. However, it is essential to evaluate the stability of these models during long-term culture and understand their differences from the *in vivo* environment. To integrate organoids into routine clinical practice for therapy decisions in a cost-effective manner, several strategies can be employed: optimizing production processes by standardizing organoid cultivation and analysis to reduce costs and time; applying automation technologies to increase yield and consistency, making organoids more suitable for clinical settings; and utilizing high-throughput screening platforms, which can significantly reduce the time and cost of drug testing, enabling large-scale application. Furthermore, combining organoids with genomic sequencing and other molecular diagnostic tools can quickly identify potential patient responses to specific therapies, offering more cost-effective personalized treatment recommendations.

Non-coding RNAs, particularly long non-coding RNAs (lncRNAs), have emerged as potential therapeutic targets due to their role in regulating the AR signaling pathway and their significant contribution to prostate cancer progression and drug resistance (100). Targeting these molecules could thus represent an innovative therapeutic strategy. To ensure the efficacy and safety of therapies targeting non-coding RNAs, comprehensive studies on biological mechanisms are essential for identifying key targets and developing effective interfering molecules. Additionally, optimized delivery systems need to be established to ensure the effectiveness and specificity of these molecules. *in vivo*, minimize off-target effects, and enhance treatment safety. For advanced CRPC patients, optimizing immune therapies like checkpoint inhibitors and vaccines such as Sipuleucel-T involves several strategies. The effectiveness of checkpoint inhibitors can be enhanced through combination therapies that amplify immune responses, such as pairing them with conventional therapies or drugs targeting metabolic pathways to counteract the immunosuppressive tumor microenvironment. Vaccines can be improved by selecting more immunogenic target antigens or using immune adjuvants to boost the immune system’s recognition capabilities. Personalized vaccine strategies, which tailor treatments based on each patient’s unique tumor antigen profile, also hold promise. The role of biomarkers is critical, as identifying and validating those predicting treatment efficacy can help in selecting the appropriate patient groups, thereby improving the overall effectiveness of immunotherapy.

## 8 Conclusion

Prostate cancer is the second most common cancer among men worldwide, with incidence rates significantly increasing with age. Although androgen deprivation therapy and second-generation AR antagonists have made some progress in improving patient prognosis, the development of CRPC and its resistance remain significant challenges. AR plays a central role in prostate cancer, with the activity of its signaling pathway directly influencing the proliferation and survival of tumor cells. The resistance of CRPC is primarily associated with AR mutations, activation of downstream signaling pathways, and metabolic reprogramming, enabling tumor cells to survive in a low-androgen environment. In recent years, new therapeutic strategies targeting AR have emerged, including novel AR antagonists, immunotherapy, and targeted therapies, providing new options. Future research should focus on developing antagonists against AR-V7, exploring AR-related biomarkers, and achieving personalized treatment plans to improve the early diagnosis and prognosis of CRPC. In summary, in-depth research into the regulatory mechanisms of AR and metabolic reprogramming will bring new hope for the treatment of prostate cancer.
